# Breaking the Cycle of Pain: The Role of Graded Motor Imagery and Mirror Therapy in Complex Regional Pain Syndrome

**DOI:** 10.3390/biomedicines12092140

**Published:** 2024-09-20

**Authors:** Danilo Donati, Paolo Boccolari, Federica Giorgi, Lisa Berti, Daniela Platano, Roberto Tedeschi

**Affiliations:** 1 Physical Therapy and Rehabilitation Unit, Policlinico di Modena, 41125 Modena, Italy; 2 Clinical and Experimental Medicine PhD Program, University of Modena and Reggio Emilia, 41121 Modena, Italy; 3 Pediatric Physical Medicine and Rehabilitation Unit, IRCCS Institute of Neurological Sciences, 40124 Bologna, Italy; 4 Department of Biomedical and Neuromotor Sciences, Alma Mater Studiorum, University of Bologna, 40126 Bologna, Italy; 5 Physical Medicine and Rehabilitation Unit, IRCCS Istituto Ortopedico Rizzoli, 40136 Bologna, Italy

**Keywords:** Complex Regional Pain Syndrome (CRPS), Graded Motor Imagery (GMI), Mirror Therapy (MT), pain management, functional rehabilitation

## Abstract

**Background:** Complex Regional Pain Syndrome (CRPS) is a chronic condition characterized by severe pain and functional impairment. Graded Motor Imagery (GMI) and Mirror Therapy (MT) have emerged as potential non-invasive treatments; this review evaluates the effectiveness of these therapies in reducing pain, improving function, and managing swelling in CRPS patients. **Methods:** A systematic review was conducted including randomized controlled trials (RCTs) that investigated GMI and MT in CRPS patients. This review was registered in PROSPERO (CRD42024535972) to ensure transparency and adherence to protocols. This review included searches of PubMed, Cochrane, SCOPUS, and Web of Science databases. Out of 81 studies initially screened, 6 were included in the final review. Studies were assessed for quality using the PEDro and RoB-2 scales. The primary outcomes were pain reduction, functional improvement, and swelling reduction. **Results:** Graded Motor Imagery (GMI) and Mirror Therapy (MT) reduced pain by an average of 20 points on the Neuropathic Pain Scale (NPS) and resulted in functional improvements as measured by the Task-Specific Numeric Rating Scale (NRS). GMI also contributed to some reduction in swelling. MT, particularly in post-stroke CRPS patients, showed significant pain reduction and functional improvements, with additional benefits in reducing swelling in certain studies. However, the included studies had small sample sizes and mixed designs, which limit the generalizability of the findings. The studies varied in sample size and design, with some risk of bias noted. **Conclusions:** Graded Motor Imagery (GMI) and Mirror Therapy (MT) have proven to be effective interventions for managing Complex Regional Pain Syndrome (CRPS), with significant improvements in pain reduction and functional recovery. These non-invasive treatments hold potential for integration into standard rehabilitation protocols. However, the small sample sizes and variability in study designs limit the generalizability of these findings. Future research should focus on larger, more homogeneous trials to validate the long-term effectiveness of GMI and MT, ensuring more robust clinical application.

## 1. Introduction

Complex Regional Pain Syndrome (CRPS) is a multifaceted clinical condition characterized by disproportionate pain relative to the extent of tissue injury, which persists well beyond the expected time of healing [1,2]. The pain associated with CRPS is frequently accompanied by a constellation of sensory, motor, and autonomic disturbances, including allodynia, hyperalgesia, vasomotor abnormalities, sudomotor changes, and trophic alterations [3,4,5,6]. These symptoms are regional, often affecting an entire limb, and do not follow the distribution of specific dermatomes or myotomes. The condition is highly disabling, with profound implications for patients’ quality of life, affecting their sleep, physical function, and social participation [7,8,9,10,11]. The etiology of CRPS is not fully understood but is often triggered by trauma, fractures, or surgical interventions, although cases of spontaneous onset have also been reported.

CRPS affects approximately 26.2 cases per 100,000 person-years, most commonly affecting individuals aged 61–70 and with a female-to-male ratio of 3:1. This is shown in studies such as the one conducted by De Mos et al. [2] in the Netherlands, which estimated an incidence of CRPS at 26.2 cases per 100,000 person-years, with a peak incidence between 61 and 70 years of age and a female-to-male ratio of approximately 3:1. Fractures are the most common precipitating event, accounting for 44% of cases, with the upper limbs being more frequently affected. The clinical presentation of CRPS can vary widely among patients and may evolve over time, complicating the development of validated and clinically useful diagnostic criteria [12,13,14,15,16].

In 1994, the International Association for the Study of Pain (IASP) named this condition “Complex Regional Pain Syndrome” and developed diagnostic criteria. Due to their low specificity, these criteria were revised in 2007 by Harden et al. [7], leading to the currently accepted “Budapest Criteria,” which have since become the standard for diagnosing CRPS [17,18,19,20].

CRPS exerts a significant burden not only on physical function but also on the mental and psychosocial well-being of patients. The condition often results in chronic pain, which is notoriously difficult to manage, leading to long-term disability and reduced quality of life [21,22,23,24,25]. Early diagnosis and intervention are crucial, as delayed treatment is associated with poorer outcomes. CRPS in its early stages is generally more responsive to treatment, while chronic CRPS is more resistant to therapeutic interventions, making it imperative to develop effective treatment strategies that can be implemented as soon as the condition is recognized.

Current treatment guidelines emphasize a multimodal approach incorporating pharmacological management, physical therapy, occupational therapy, and psychological support. Among the physiotherapeutic interventions, manual therapy [26], therapeutic exercises, and progressive loading regimens are commonly recommended [27,28,29]. In addition, electrotherapy modalities such as transcutaneous electrical nerve stimulation (TENS), ultrasound, and laser therapy have been explored [30]. However, increasing attention is being given to interventions that target cortical reorganization and sensorimotor integration, such as Graded Motor Imagery (GMI) and Mirror Therapy (MT).

GMI and MT were selected for this review due to their ability to target the cortical reorganization and sensorimotor integration dysfunctions associated with CRPS, mechanisms thought to underlie the condition’s chronicity [31,32,33].

GMI and MT are based on the premise that CRPS and other chronic pain conditions may involve maladaptive changes in cortical representations. These therapies aim to normalize these changes and reduce pain by gradually engaging the motor cortex in a non-threatening manner. GMI, in particular, involves a sequential approach that begins with laterality training, progresses to motor imagery, and culminates in mirror therapy, where the patient observes the reflected image of the unaffected limb performing movements, creating the illusion that the affected limb is moving without pain [34,35,36,37,38,39,40,41,42]. The effectiveness of GMI and MT [43,44,45,46,47,48] in treating CRPS has been the subject of several studies, with many reporting statistically significant improvements in pain and function. However, the evidence is not without limitations. Studies often suffer from small sample sizes, heterogeneity in patient populations, and variations in treatment protocols, which complicate the interpretation of results and limit the generalizability of findings.

## 2. Methods

The present scoping review was conducted following the JBI methodology [49,50]. The JBI methodology ensures a rigorous approach to summarizing evidence by outlining clear inclusion criteria, while the PRISMA-ScR checklist ensures transparent and complete reporting of all aspects of the review process, for scoping reviews. The Preferred Reporting Items for Systematic reviews and Meta-Analyses extension for Scoping Reviews (PRISMA-ScR) [51,52] Checklist for reporting was used.

The protocol was published in PROSPERO (International Prospective Register of Systematic Reviews) under registration number CRD42024535972.

### 2.1. Review Question

We formulated the following research question: “What is the efficacy of Graded Motor Imagery (GMI) and Mirror Therapy (MT) in reducing pain, functional improvement, and decreasing swelling in patients with Complex Regional Pain Syndrome (CRPS) compared to conventional rehabilitation treatments or other therapeutic modalities?”.

### 2.2. Eligibility Criteria

Studies were eligible for inclusion if they met the following Population, Concept, and Context (PCC) criteria.

Population (P): Studies were eligible if they involved individuals diagnosed with Complex Regional Pain Syndrome (CRPS), including both CRPS Type I and Type II, across any age or demographic. Inclusion required a clear diagnosis of CRPS based on either the International Association for the Study of Pain (IASP) criteria or the Budapest Criteria, strictly applied. Studies involving participants across all age ranges and genders were included, though future reviews should consider how age or gender may influence outcomes.

Concept (C): The focus was on evaluating the effectiveness of Graded Motor Imagery (GMI) and Mirror Therapy (MT). These interventions target the cortical and sensorimotor dysfunctions in CRPS. Studies had to compare GMI and/or MT to conventional rehabilitation, placebo, or no treatment, with key outcomes being pain reduction, functional improvement, and swelling reduction.

Context (C): Eligible studies were conducted in any healthcare or research setting, including hospitals, clinics, and rehabilitation centers. There were no restrictions on geographical location or language, as long as the studies met quality standards and provided relevant data. The context also required adequate follow-up to assess the long-term effects of the interventions. Adequate follow-up was defined as a minimum of 6 months post-intervention to assess the long-term effects of GMI and MT.

### 2.3. Exclusion Criteria

Studies that did not meet the specific PCC criteria were excluded.

### 2.4. Search Strategy

An initial limited search of MEDLINE was performed through the PubMed interface to identify articles on the topic, and then the index terms used to describe the articles were used to develop a comprehensive search strategy for MEDLINE. The search strategy, which included all identified keywords and index terms, was adapted for use in Cochrane Central, Scopus, PEDro, and Web of Science. In addition, grey literature and reference lists of all relevant studies were also searched. Searches were conducted on 31 July 2024 with no date limitation.

PubMed: (complex regional pain syndrome OR sudeck atrophy OR reflex sympathetic dystrophy) AND (graded motor imagery OR mirror therapy).

Scopus: (TITLE-ABS-KEY (“complex regional pain syndrome” OR “sudeck atrophy” OR “reflex sympathetic dystrophy”) AND TITLE-ABS-KEY (“graded motor imagery” OR “mirror therapy”)).

Cochrane: (“complex regional pain syndrome” OR “sudeck atrophy” OR “reflex sympathetic dystrophy”) AND (“graded motor imagery” OR “mirror therapy”).

Web of Science: TS = (“complex regional pain syndrome” OR “sudeck atrophy” OR “reflex sympathetic dystrophy”) AND TS = (“graded motor imagery” OR “mirror therapy”).

Pedro: (complex regional pain syndrome* AND graded motor imagery) OR (complex regional pain syndrome* AND mirror therapy).

### 2.5. Study Selection

The process described involves a systematic approach to selecting studies for a scoping review. Initially, search results were collected and refined using Zotero, with duplicates removed. The screening involved two levels: title and abstract review, followed by full-text assessment, both conducted independently by two authors with discrepancies resolved by a third. The selection adhered to the PRISMA 2020 guidelines, ensuring transparency and reliability. This rigorous methodology aimed to identify relevant articles that directly address the research question, maintaining a comprehensive and systematic approach in the review process.

### 2.6. Data Extraction and Data Synthesis

Data extraction for the scoping review was performed using a form based on the JBI tool, capturing crucial details like authorship, publication country and year, study design, patient characteristics, outcomes, interventions, procedures, and other relevant data. Descriptive analyses of this data were conducted, with results presented numerically to show study distribution. The review process was clearly mapped for transparency, and data were summarized in tables for easy comparison and understanding of the studies’ key aspects and findings.

## 3. Results

**Figure 1:**
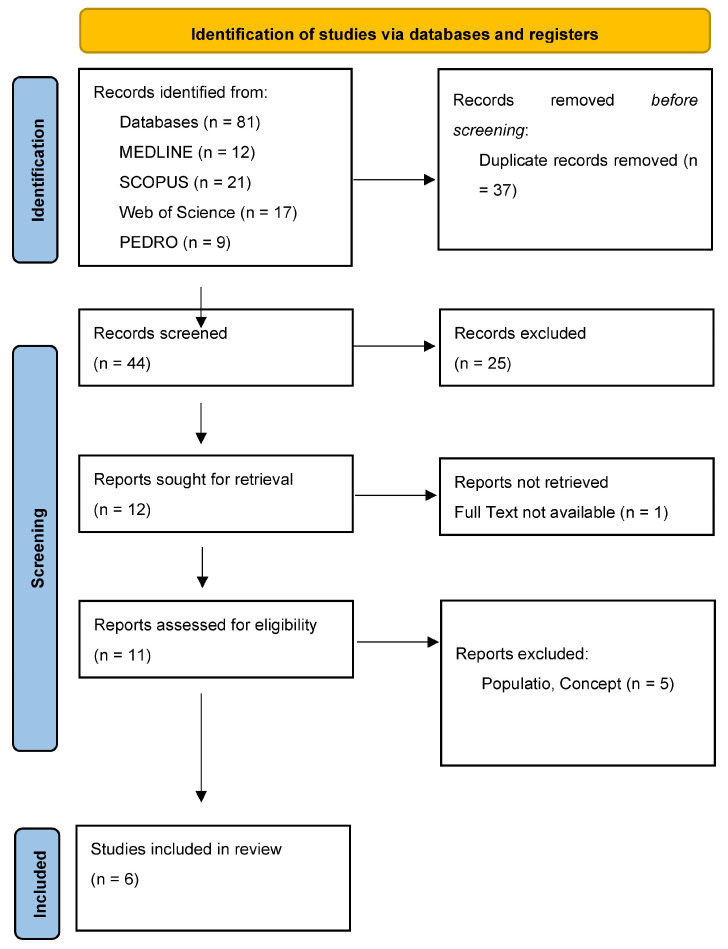
Preferred reporting items for systematic reviews and meta-analyses 2020 (PRISMA) flow-diagram.

As presented in the PRISMA 2020-flow diagram (Figure 1), from 81 records identified by the initial literature searches, 75 were excluded and 6 articles were included (Table 1, Table 2 and Table 3). The quality of the studies was assessed with a PEDro scale (Table 4) and ROB2 (Table 4).

### 3.1. Pain Reduction

Across the six studies, pain reduction emerged as a significant outcome, particularly with the use of Graded Motor Imagery (GMI) and Mirror Therapy (MT). Moseley’s series of studies, conducted in 2004, 2005, and 2006 [45,46,47], consistently demonstrated that GMI leads to substantial reductions in pain levels among patients with CRPS. In the 2004 study, for example, patients experienced a marked decrease in pain, as reflected by a 20-point reduction on the Neuropathic Pain Scale (NPS). This decrease was not only statistically significant but also clinically meaningful, indicating a notable improvement in the patients’ daily pain experience.

Similarly, the 2005 study by Moseley further reinforced these findings, showing that GMI outperformed conventional pharmacological treatments in reducing pain. The patients in the GMI group reported a greater reduction in pain, highlighting the therapy’s potential as an alternative or adjunct to traditional pain management strategies. By 2006, Moseley’s research had expanded to show that the pain relief provided by GMI was sustained over a six-month follow-up period, underscoring the long-term benefits of this intervention.

Mirror Therapy (MT) also proved effective in reducing pain, particularly in post-stroke CRPS patients. Cacchio et al. (2009) [37] reported significant pain reduction in patients receiving MT, with these effects lasting up to 24 weeks post-intervention. This suggests that MT could be a valuable tool in managing chronic pain in CRPS, particularly in populations where other treatments might be less effective. Similarly, Vural et al. (2016) [38] found that MT led to significant decreases in pain, supporting the notion that visual-motor feedback can play a crucial role in pain modulation. Sarkar et al. (2017) [39] expanded on these findings by demonstrating that both GMI and MT could effectively reduce pain during rest and movement, making them versatile options in the treatment of CRPS-related pain.

### 3.2. Functional Improvement

Functional improvement was another key outcome observed across the studies, particularly with GMI and MT. Moseley’s 2004 study [45] showed that GMI not only reduced pain but also significantly improved patients’ ability to perform daily activities. This improvement was measured using the Task-Specific Numeric Rating Scale (NRS), where patients reported a higher level of functional improvement post-intervention. The benefits were not just immediate; they were also sustained, as seen in the 2005 [46] and 2006 [47] studies, where functional gains were maintained at follow-ups, including a significant enhancement in the ability to perform specific tasks with less pain and greater ease.

Mirror Therapy (MT) also demonstrated strong results in enhancing function, particularly in post-stroke patients with CRPS. Cacchio et al. (2009) [37] found that MT led to significant improvements in motor function, as measured by the Wolf Motor Function Test (WMFT), and in the quality of movement, assessed by the Motor Activity Log—Quality of Movement (MAL-QOM). These improvements suggest that MT can be particularly beneficial in neurorehabilitation, where restoring motor function is often a primary goal. Vural et al. (2016) [38] also reported significant functional gains in hand function, as indicated by the Fugl–Meyer Assessment (FMA) scores, reinforcing the role of MT in comprehensive stroke rehabilitation. Sarkar et al. (2017) [39] further supported these findings, demonstrating that both GMI and MT led to significant functional improvement in CRPS patients, with improvements noted in standardized assessments like the FMA and Task-Specific NRS.

### 3.3. Swelling Reduction

Swelling reduction, a less commonly addressed but equally important outcome in CRPS treatment, was significantly impacted by both GMI and MT. Moseley (2004) [45] reported a noteworthy reduction in limb swelling following GMI intervention, with the circumference of the affected limb decreasing by approximately 1.0 cm. This finding is particularly important as it highlights GMI’s potential to address not just the sensory and functional symptoms of CRPS, but also the physical manifestations like edema [53].

Sarkar et al. (2017) [39] also observed similar benefits with both GMI and MT, where patients experienced a decrease in swelling, further supporting the effectiveness of these therapies in managing the broader spectrum of CRPS symptoms. The reduction in edema, as measured through circumferential assessments, indicates that these interventions can help in alleviating some of the more distressing physical symptoms of CRPS, contributing to an overall improvement in patient well-being.

## 4. Discussion

The findings from this review underscore the potential of Graded Motor Imagery (GMI) and Mirror Therapy (MT) as effective interventions for managing Complex Regional Pain Syndrome (CRPS). GMI showed greater improvement in functional outcomes, whereas MT was particularly effective in reducing swelling. Both therapies demonstrated significant benefits across multiple outcomes, including pain reduction, functional improvement, and swelling reduction [37,38,39,45,46,47]. These results align with the growing body of literature that suggests CRPS, a condition that is notoriously difficult to treat, may be effectively managed through neuroplasticity-driven interventions [54,55]. One of the most striking findings from this review is the consistent effectiveness of GMI in reducing pain, as shown in the studies by Moseley. The sustained pain relief observed across multiple studies suggests that GMI addresses the underlying mechanisms of CRPS rather than merely providing symptomatic relief. This long-term effectiveness is particularly significant given the chronic nature of CRPS and the challenges associated with managing persistent pain in these patients [36,56,57]. GMI’s impact on functional improvement further emphasizes its role in comprehensive rehabilitation programs, offering patients not only relief from pain but also a restoration of function, which is crucial for improving quality of life [58,59]. Mirror Therapy also showed considerable promise, especially in post-stroke CRPS patients. MT is believed to correct distorted cortical representations by providing visual feedback, effectively reestablishing normal sensory and motor processing. Neuroimaging studies have shown that MT can modulate cortical excitability, which is associated with pain reduction in CRPS patients. The studies reviewed indicate that MT can lead to significant improvements in both pain and functional improvement, with benefits observed well into the follow-up periods. This suggests that MT, similar to GMI, may induce lasting neuroplastic changes that help reestablish more normal sensory and motor processing. The mechanism by which MT achieves these effects likely involves the correction of distorted cortical representations of the affected limb, which are thought to contribute to the chronicity of CRPS symptoms [29,60,61,62,63]. The success of MT in this context highlights its potential as a non-invasive, cost-effective therapy that could be easily integrated into standard rehabilitation protocols [64,65,66]. However, while the findings are promising, it is important to recognize the limitations of this review and the included studies. One notable limitation is the small sample sizes in many of the studies, which raises concerns about the generalizability of the findings. Small sample sizes can lead to an overestimation of treatment effects and reduce the statistical power of the studies, potentially biasing the results. Moseley (2004) showed a significant reduction in pain, but the small sample size (13 participants) may have led to an overestimation of the treatment effect. This issue is compounded by the variability in study designs, interventions, and outcome measures across the included studies, which makes it difficult to directly compare results or draw definitive conclusions about the relative efficacy of GMI versus MT. Another limitation is the potential for bias in the included studies, as evidenced by the quality assessments using the PEDro and RoB-2 scales. While most studies demonstrated a low risk of bias, particularly in randomization and outcome measurement, there were some concerns related to deviations from intended interventions and selection of reported results. These biases could influence the reported effectiveness of the interventions and must be considered when interpreting the findings. Additionally, the heterogeneity of the patient populations, particularly in terms of the duration and severity of CRPS, may have influenced the outcomes. CRPS is a highly variable condition, and its presentation can differ significantly from one patient to another. The reviewed studies included both early and chronic CRPS cases, as well as patients with varying etiologies (e.g., post-trauma vs. post-stroke). This variability makes it challenging to generalize the findings to all CRPS patients and suggests that the effectiveness of GMI and MT may vary depending on the stage and underlying cause of the condition [36,45,47]. Furthermore, the review was limited by the scope of available literature. Despite an exhaustive search strategy, there may be relevant studies that were not identified or included, particularly those published in languages other than English or those with negative results that were less likely to be published. The inclusion criteria, while necessary for maintaining the quality of the review, may have also excluded valuable data from less rigorous but still informative studies. Finally, the long-term effectiveness and practicality of implementing GMI and MT in diverse clinical settings remain areas that require further investigation. Although some studies included follow-up periods, the sustainability of the observed benefits over months or years has not been fully established. Additionally, the feasibility of these interventions in routine clinical practice, particularly in settings with limited resources, is an important consideration that was not fully addressed by the reviewed studies. While GMI and MT show significant promise for managing CRPS, particularly in reducing pain and functional improvement, more research is needed to confirm these findings across larger, more diverse patient populations and to explore the long-term benefits and practical implementation of these therapies.

### 4.1. Study Selection and Publication Year Distribution

The included studies spanned from 2004 to 2023 with none published within the past five years. Our search yielded 44 non-duplicated records, but only six met the inclusion criteria. This reflects the specific challenges of conducting randomized controlled trials (RCTs) on Complex Regional Pain Syndrome (CRPS). CRPS is a relatively rare and heterogeneous condition, making it difficult to recruit sufficiently large and diverse populations. Many of the recent studies either lacked control groups or were not designed as RCTs, which are necessary for maintaining the rigor of this review.

### 4.2. Challenges in Conducting RCTs in CRPS

Conducting RCTs for CRPS presents unique difficulties, including low incidence rates, high variability in patient presentations, and ethical concerns around withholding treatment in control groups. Moreover, CRPS is often a chronic condition that progresses differently across individuals, making it challenging to design standardized trials that fit diverse patient populations. These factors likely contribute to the decrease in qualifying studies in recent years.

Future studies should aim to address the limitations identified in this review, including small sample size, potential bias, and patient heterogeneity, to provide more robust and generalizable evidence on the efficacy of GMI and MT in the treatment of CRPS. Future research should also focus on large-scale longitudinal studies to assess the duration of the therapeutic benefits of GMI and MT, especially beyond the 6-month follow-up period reported in existing studies.

## 5. Clinical Practice Implications

The findings from this review suggest that Graded Motor Imagery (GMI) and Mirror Therapy (MT) can be valuable tools in the management of Complex Regional Pain Syndrome (CRPS) within clinical settings. These interventions offer non-invasive, cost-effective options that can be integrated into standard rehabilitation protocols to address the chronic pain and functional limitations associated with CRPS. For clinicians, the use of GMI and MT should be considered early in the treatment plan for CRPS patients, particularly given the evidence of their effectiveness in reducing pain and functional improvement. GMI can be incorporated into daily therapy sessions, focusing on gradual, sequential activation of motor cortical areas, while MT can be easily administered with minimal equipment, making it accessible even in resource-limited settings. Both therapies can be personalized to fit the patient’s specific condition and progress, allowing for flexible treatment plans that adapt to individual needs. Given the chronic nature of CRPS, these interventions also offer the advantage of being sustainable and feasible for long-term use, either as standalone treatments or in combination with other modalities.

## 6. Conclusions

The studies reviewed show an average pain reduction of 20 points on the Neuropathic Pain Scale and a significant improvement in functional capacity. Both interventions have shown significant potential in reducing pain and functional improvement outcomes, offering non-invasive and cost-effective options for patients struggling with this challenging condition. Compared to conventional rehabilitation techniques, GMI and MT offer more focused interventions targeting cortical reorganization, providing significant improvements in both pain reduction and functional recovery. Despite the positive findings, the review also identified the need for further research, particularly studies with larger sample sizes and more diverse populations, to confirm these results and establish long-term efficacy.

## Figures and Tables

**Table 1:** Main characteristics of included studies. Summary of six Randomized Controlled Trials (RCTs) investigating the efficacy of Graded Motor Imagery (GMI) and Mirror Therapy (MT) in patients with Complex Regional Pain Syndrome (CRPS).

Author(s)	Title	Year	Methods	Results	Outcomes Achieved
Moseley [45]	Graded motor imagery is effective for long-standing complex regional pain syndrome	2004	Single-blind, randomized controlled trial with crossover. 13 participants with CRPS Type I.	Significant reduction in pain and swelling post-GMI. Sustained functional improvement.	Pain reduction (NPS), decrease in swelling (circumference measurement), improved task-specific function (NRS).
Moseley [46]	Graded motor imagery for pathologic pain	2005	Parallel-group, single-blind RCT with 3 arms. 21 participants with CRPS Type I.	Pain reduction was greater in the GMI group compared to control groups. Functional improvements also noted.	Pain reduction (NPS), functional improvement (task-specific NRS).
Moseley [47]	Graded motor imagery for pathologic pain: A randomized controlled trial	2006	Parallel-group, single-blind RCT with 2 arms. 37 participants with CRPS Type I.	Significant pain reduction and improvement in function maintained at 6-month follow-up.	Pain reduction (VAS), functional improvement (task-specific NRS), maintained at follow-up.
Cacchio et al. [37]	Mirror therapy for functional improvement outcome in patients with post-stroke CRPS	2009	Single-blind RCT, 48 post-stroke CRPS patients, 2 groups: mirror therapy vs. placebo mirror therapy.	Mirror therapy group showed significant improvement in pain and function compared to placebo.	Pain reduction (VAS), functional improvement (WMFT), and quality of movement (MAL-QOM).
Vural et al. [38]	Effectiveness of mirror therapy on pain and hand function in stroke patients with CRPS	2016	Single-blind RCT with 2 arms. 30 post-stroke CRPS patients.	Mirror therapy resulted in significant pain reduction and improvement in hand function.	Pain reduction (VAS), improved hand function (FMA hand-wrist subsection).
Sarkar et al. [39]	Effect of graded motor imagery and mirror therapy on pain and functional outcome in CRPS	2017	Single-blind RCT with 3 arms. 30 participants with CRPS Type I.	Both GMI and MT groups showed significant reductions in pain and functional improvements compared to placebo.	Pain reduction (NPRS), decreased swelling, functional improvement (FMA, task-specific NRS).

Legend: CRPS: Complex Regional Pain Syndrome, FMA: Fugl–Meyer Assessment, GMI: Graded Motor Imagery, MAL-QOM: Motor Activity Log—Quality of Movement, MT: Mirror Therapy, NPRS: Numeric Pain Rating Scale, NPS: Neuropathic Pain Scale, NRS: Numeric Rating Scale, RCT: Randomized Controlled Trial, VAS: Visual Analogue Scale, WMFT: Wolf Motor Function Test.

**Table 2:** Baseline Population Characteristics. Baseline population characteristics of six Randomized Controlled Trials (RCTs) investigating Graded Motor Imagery (GMI) and Mirror Therapy (MT) in patients with Complex Regional Pain Syndrome (CRPS).

Author(s)	Sample Size	Age (Mean ± SD)	Gender (M/F)	Duration of CRPS (Mean ± SD)	Type of CRPS
Moseley [45]	13	35 ± 15 years	02-nov	6 months	Type I
Moseley [46]	21	36 ± 8 years	giu-15	12 ± 6 months	Type I
Moseley [47]	37	45 ± 14 years	nov-26	14 ± 10 months	Type I
Cacchio et al. [37]	48	57.9 ± 9.9 years	nov-37	6 months	Post-stroke CRPS
Vural et al. [38]	30	68.9 ± 10.5 years	15/15	Not reported	Post-stroke CRPS
Sarkar et al. [39]	30	Not reported	Not reported	Not reported	Type I

**Table 3:** Intervention characteristics. Intervention characteristics of six Randomized Controlled Trials (RCTs) investigating Graded Motor Imagery (GMI) and Mirror Therapy (MT) in patients with Complex Regional Pain Syndrome (CRPS).

Author(s)	Intervention	Duration of Intervention	Comparator	Follow-Up
Moseley [45]	Graded Motor Imagery (GMI)	6 weeks (daily sessions)	Pharmacological treatment	6 weeks and 12–18 weeks post-intervention
Moseley [46]	Graded Motor Imagery (GMI)	6 weeks (daily sessions)	Pharmacological treatment	6 weeks and 12 weeks post-intervention
Moseley [47]	Graded Motor Imagery (GMI)	6 weeks (daily sessions)	Pharmacological treatment	6 weeks and 24 weeks post-intervention
Cacchio et al. [37]	Mirror Therapy (MT)	4 weeks (5 sessions/week)	Placebo mirror therapy	4 weeks and 24 weeks post-intervention
Vural et al. [38]	Mirror Therapy (MT)	4 weeks (5 sessions/week)	Conventional rehabilitation	No follow-up
Sarkar et al. [39]	Graded Motor Imagery (GMI) and Mirror Therapy (MT)	4 weeks (twice daily sessions)	Placebo mirror therapy	No follow-up

**Table 4:** Quality assessment using PEDro and RoB-2 scales. Quality assessment of six Randomized Controlled Trials (RCTs) investigating Graded Motor Imagery (GMI) and Mirror Therapy (MT) in patients with Complex Regional Pain Syndrome (CRPS) using the PEDro and RoB-2 scales.

Author(s)	PEDro Score (Out of 10)	RoB-2: Bias Due to Randomization	RoB-2: Bias Due to Deviations from Intended Interventions	RoB-2: Bias Due to Missing Outcome Data	RoB-2: Bias in Measurement of the Outcome	RoB-2: Bias in Selection of the Reported Result	Overall RoB-2 Judgment
Moseley (2004) [45]	6	Low	Low	Low	Low	Low	Low
Moseley (2005) [46]	6	Low	Low	Low	Low	Low	Low
Moseley (2006) [47]	5	Low	Low	Low	Low	Low	Low
Cacchio et al. (2009) [37]	7	Low	Low	Low	Low	Low	Low
Vural et al. (2016) [38]	6	Low	Low	Low	Low	Low	Low
Sarkar et al. (2017) [39]	4	Some concerns	High	Low	Low	Low	High

Legend: PEDro Score: Physiotherapy Evidence Database Score, RoB-2: Risk of Bias 2 Tool.

## Data Availability

No new data were created or analyzed in this study. Data sharing is not applicable to this article.
